# Proper Food for Consumptives

**Published:** 1889-12

**Authors:** 


### PROPER FOOD FOR CONSUMPTIVES.

Experience has shown that consumptives can assimilate a quantity of food far in excess of the needs of the healthy organism. This excessive quantity should be supplied; though resort to the stomach tube, as in Debove's experiments, can be reserved for special cases.

Meat, principally beef, with milk, fish, eggs, leguminous vegetables and greens should form the bulk of the dietary; with the addition of fat, in large quantities, in the form of cream, butter, oil dressing for salads, or, if necessary, cod-liver oil or oleaginous inunctions. Alcohol is a food in consumption. It should be combined with malt, milk, glycerin, or cod-liver oil. Not more than three, at most four, hours, except during sleep, should be allowed to elapse without taking food. Milk punch on going to bed, and a glass of wine or spirits with liquid peptonoids in case of waking during the night, are strongly urged. Hot water before meals, if necessary, to prepare the alimentary tract for the digestion and absorption of food, and the free drinking of water to wash out waste products are essential to success. Predigested foods may be employed with great benefit, especially peptonized milk and beef pep tonoids.

Proper alimentary diet being secured through over-feeding, and the digestive tract and its annex being kept in good condition, proper respiratory diet must be supplied. Open air life, exercise on horseback or bicycle, voluntary respiratory gymnastics, may suffice when the patient is still strong enough. In the majority of cases there must be some artificial aid. The inhalation of compressed air, with or without expiration into rarefied air is the best means. Apparatus should be simple and cheap, so that the patient can use it at home.

The effects of pneumatic measures are to increase the volume of re. spired air, open up unused air cells, facilitate expulsion of pathological products, increase gaseous exchange, facilitate absorption of oxygen by the hæmoglobin, while at the same time the volume of blood and the activity of circulation in the lungs is increased, bringing more corpus. cles into contact with the increased volume of oxygen. Penetration of blood into the tissues, tissue respiration, and production of lymph are also increased. Thus local and general nutrition are powerfully stimulated, and an improved respiratory habit and increased respiratory and circulatory power are gained. The products of digestion are properly oxidized and utilized, immature tissue is destroyed and expelled. The promotion of sleep is one of the most marked effects.

The physician thus puts his patient into a condition where natural powers complete the cure. and bacilli need not be feared.

